# A drone-based prototype technique for monitoring soil degassing at active volcanic craters

**DOI:** 10.1186/s40623-025-02303-9

**Published:** 2025-10-23

**Authors:** Társilo Girona, Jason Williams, James Copple, Matthew Westhoff, Kyriaki Drymoni, Noé García-Martínez, David Benavente, Conor A. Bacon, Maarten de Moor, Einat Lev

**Affiliations:** 1 Geophysical Institute, University of Alaska Fairbanks, Fairbanks, AK USA; 2 Geosciences Barcelona (GEO3BCN), CSIC, Barcelona, Spain; 3 Alaska Center for Unmanned Aircraft Systems Integration (ACUASI), Geophysical Institute, University of Alaska Fairbanks, Fairbanks, AK USA; 4 Earth and Environmental Sciences, Ludwig-Maximilians-Universität in Munich, Munich, Germany; 5 Jet Propulsion Laboratory, California Institute of Technology, Pasadena, CA USA; 6 Earth and Environmental Sciences Department, University of Alicante, Alicante, Spain; 7 Lamont-Doherty Earth Observatory, Columbia University, New York, NY USA; 8 Observatorio Vulcanológico y Sismológico de Costa Rica, Universidad Nacional, Heredia, Costa Rica; 9 Department of Earth and Planetary Sciences, University of New Mexico, Albuquerque, NM USA

**Keywords:** Soil degassing, Soil properties, Remotely Piloted Aircraft System (RPAS), Poás volcano, Eruption, Volcano monitoring, Risk

## Abstract

Developing techniques to monitor volcanic activity from safe distances is crucial for advancing scientific knowledge while protecting the safety of field personnel. One of the most demanding tasks in this context is the measurement of soil gas emissions, which offer valuable insights into fluid migration through the shallow crust and act as an early indicator of volcanic unrest and potential eruptive activity. Traditional soil degassing measurements commonly require two operators to be physically present with the instrument, sometimes exposing them to hazardous conditions. In this study, we present a new method for performing soil degassing measurements from a safe distance, using a customized Remotely Piloted Aircraft System (RPAS). This drone-based approach was designed to carry out accumulation chamber measurements in hazardous or otherwise inaccessible areas. We tested the system at four locations around the active crater of Poás Volcano in Costa Rica, where we collected data on CO_2_ and H_2_O fluxes, along with soil temperature and moisture. Our results reveal spatial variability in gas emissions and surface conditions across the study sites. A site located on the crater rim (Site 1) showed the highest CO_2_ and H_2_O fluxes, indicating active gas release possibly associated with structural features. A second site, located within the crater (Site 2), exhibited elevated H_2_O flux without detectable CO_2_, suggesting localized processes related to moisture transport. Our experiment on another crater site (Site 3) produced a complete and high-quality dataset, demonstrating the operational success of the method. In contrast, measurements at the last crater site (Site 4) were affected by chamber sealing issues and potentially by the influence of volcanic gas plumes. While the experiment faced several challenges, including imperfect ground-sensor contact as well as occasional telemetry interruptions, it successfully demonstrated the feasibility of using drones for soil degassing surveys. Based on these findings, we identify specific areas for improvement and propose future directions to enhance the system reliability and performance. Overall, this method offers a promising tool for extending soil gas measurements to hazardous or hard-to-reach environments, contributing to safer and more comprehensive monitoring of active volcanic systems.

**Graphical Abstract:**

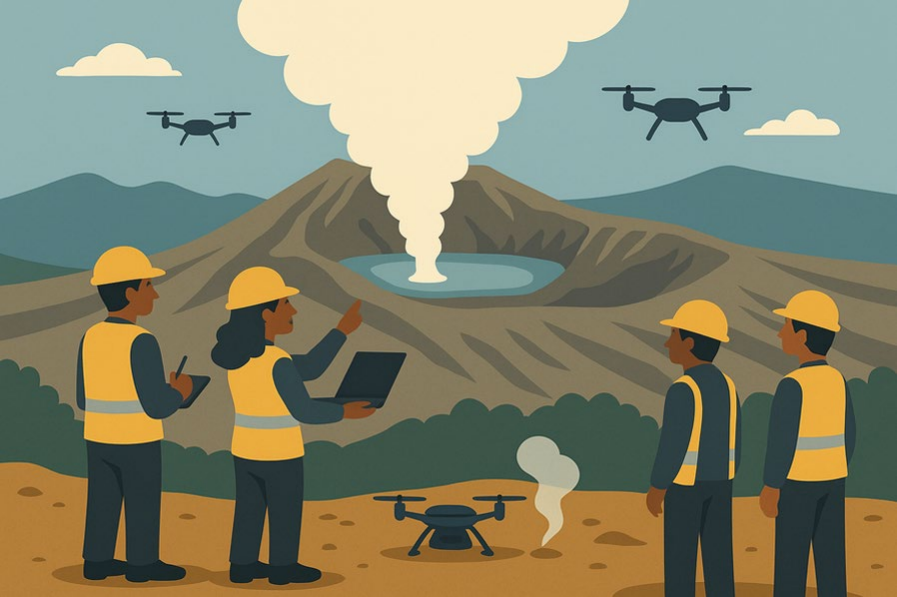

**Supplementary Information:**

The online version contains supplementary material available at 10.1186/s40623-025-02303-9.

## Introduction

Soil emissions of volcanic-hydrothermal gases provide valuable insights into subsurface processes at active volcanoes. These emissions have proven crucial not only for the early detection of volcanic unrest (e.g., Baubron et al. 1991; Pérez et al. 2012; Liuzzo et al. 2013; Parks et al. 2013; Melián et al. 2014; Cardellini et al. 2017), but also for identifying zones of elevated permeability (e.g., faults, rift systems, and stratigraphic discontinuities) and for tracing shallow fluid circulation pathways in the crust (e.g., Chiodini et al. 1996, 2008; Toutain et al. 2002; Cartagena et al. 2004; Giammanco et al. 2010; Carapezza et al. 2011; Liuzzo et al. 2015). To improve our understanding of volcanic and hydrothermal systems, it is therefore essential to advance soil degassing measurement techniques (e.g., Allard et al. 1991; Chiodini et al. 1998). Among the gases released through the soil, CO_2_ is the most widely measured species, with both direct and indirect methods available for quantifying its flux (Chiodini et al. 1998). Indirect approaches involve estimating CO_2_ concentration at different depths, while direct measurements, typically conducted at a fixed depth, are often favored due to their broader applicability and reduced methodological limitations. In turn, direct methods are commonly categorized into dynamic and static techniques, each with distinct operational principles and advantages.

Dynamic techniques estimate soil CO_2_ concentrations by sampling at fixed depths a known airflow containing a mixture of atmospheric air and soil-emitted CO_2_, which is transported primarily by diffusion and/or advection (Chiodini et al. 1998). This mixture is collected using inverted chambers (Kucera and Kirkham 1971) or pipe-and-tube systems (Gurrieri and Valenza 1988; Finizola et al. 2006), then pumped and analyzed using infrared spectrophotometers (Liuzzo et al. 2015). Owing to their robustness and reduced sensitivity to environmental variability, these techniques are well-suited for real-time monitoring and have been incorporated into operational networks at active volcanoes (e.g., EtnaGAS network and Stromboli stations; Liuzzo et al. 2013; Laiolo et al., 2016). However, a known limitation of dynamic methods is their tendency to overestimate fluxes compared to static chamber techniques (Witkamp and Frank 1969; Tonani and Miele 1991; Carapezza and Granieri 2004).

Static techniques do not rely on a known airflow and include two primary approaches: (i) the in situ alkaline solution or soda-lime method, which captures CO_2_ within a sealed chamber using a chemical absorbent (Cropper et al. 1985); and (ii) the accumulation chamber method (also known as the “zero-depth at time-zero” method), which continuously measures the rate of CO_2_ concentration increase inside a closed chamber placed on the soil surface, typically using an infrared spectrometer (Tonani and Miele 1991; Chiodini et al. 1996, 1998; Cartagena et al. 2004). Since its initial development, the accumulation chamber method has been extensively validated, demonstrating high reproducibility across different soil types (deviation < 10%), strong agreement with flowmeter-based measurements (< 13%), and excellent consistency with laboratory results (< 5%), with additional field tests confirming its reliability (Chiodini et al. 1998). Owing to its operational simplicity, rapid data acquisition, and ability to provide accurate results without significant soil or atmospheric corrections, the accumulation chamber method has become the state-of-the-art approach for measuring soil CO_2_ fluxes in volcanology (e.g., Chiodini et al. 2008; Carapezza and Granieri 2004; Carapezza et al. 2011; Parks et al. 2013; Nelson et al. 2024).

Soil degassing campaigns using the accumulation chamber method have proven valuable for improving short-term eruption forecasting (e.g., Giammanco et al. 2010; Carapezza et al. 2011; Liuzzo et al. 2013, 2015). However, their effectiveness relies on in situ measurements, requiring volcanologists to operate in remote, difficult-to-access, and hazardous environments, particularly during periods of elevated volcanic activity. Under such conditions, researchers are frequently exposed to dangerous concentrations of acidic gases such as CO_2_, SO_2_, H_2_S, HF, HCl, and sulfate aerosols (SO_4_^2^⁻), which can reach critical levels in areas surrounding active volcanoes (Hansell et al. 2006; Carapezza et al. 2011). Most critically, this exposure significantly increases the risk to personnel in the event of a sudden eruption (Aramaki et al. 1994).

Minimizing the risks associated with volcano research has become an increasing priority for the scientific community (e.g., James et al. 2020; Román et al. 2022). In response, airborne techniques have gained considerable traction, particularly the use of Remotely Piloted Aircraft Systems (RPAS), also referred to as Unmanned Aerial Vehicles (UAVs), Unmanned Aircraft Systems (UAS), or drones (e.g., Cress et al. 2015; Gomez and Purdie 2016). Compared to traditional ground-based methods, RPAS offer multiple advantages, including cost efficiency, operational flexibility, and the capacity to obtain data at high spatial and temporal resolutions (James et al. 2020). Additionally, RPAS can carry multiple instruments at once (e.g., visible-light cameras, thermal infrared cameras, gas sensors), providing context for measurements, increasing survey efficiency, and helping mitigate uncertainties (e.g., Agüera-Vega et al. 2017; Antoine et al. 2020; Liu et al. 2020). For example, drones have proven to be highly effective platforms for acquiring in situ measurements of fumarolic gases during volcanic field campaigns (e.g., Stix et al. 2018; Jordan 2019; Liu et al. 2020; Ericksen et al. 2022; Schmid et al. 2023; Fischer et al. 2024). However, their use has not yet been extended to soil degassing surveys.

**Fig. 1:**
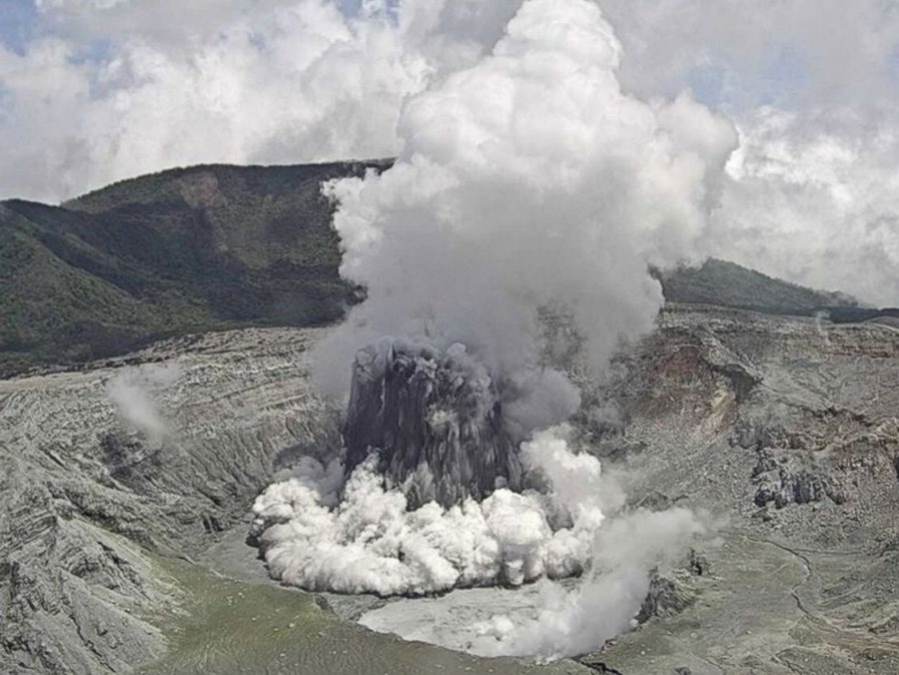
Phreatic eruption at Poás Volcano on 9 March 2025. This eruption produced an eruptive plume of ~100 m high through the crater lake. Image captured by the Observatorio Vulcanológico y Sismológico de Costa Rica (OVSICORI) webcam. Escalating volcanic activity during early 2025 led to the closure of Poás Volcano National Park, restricting direct crater access and emphasizing the necessity for RPAS-based gas measurement techniques

In this study, we present the first test of a prototype that integrates the accumulation chamber method with an RPAS to remotely measure soil gas fluxes (CO_2_ and also H_2_O) as well as related soil properties from a safe distance. In particular, we report the results of a field experiment conducted at Poás Volcano (Costa Rica) in March 2025, designed to evaluate the prototype’s performance, assess its accuracy and reliability, and identify potential improvements to optimize the methodology. During early 2025, Poás exhibited escalating activity (Fig. 1), rendering crater access unsafe for scientists and underscoring the necessity for an RPAS-based gas measurement technique. This technique represents a step forward in soil degassing campaigns, enhancing the safety, efficiency, and accessibility of volcanic gas monitoring for the long-term surveillance of active volcanoes.

## Method

### System description

**Fig. 2:**
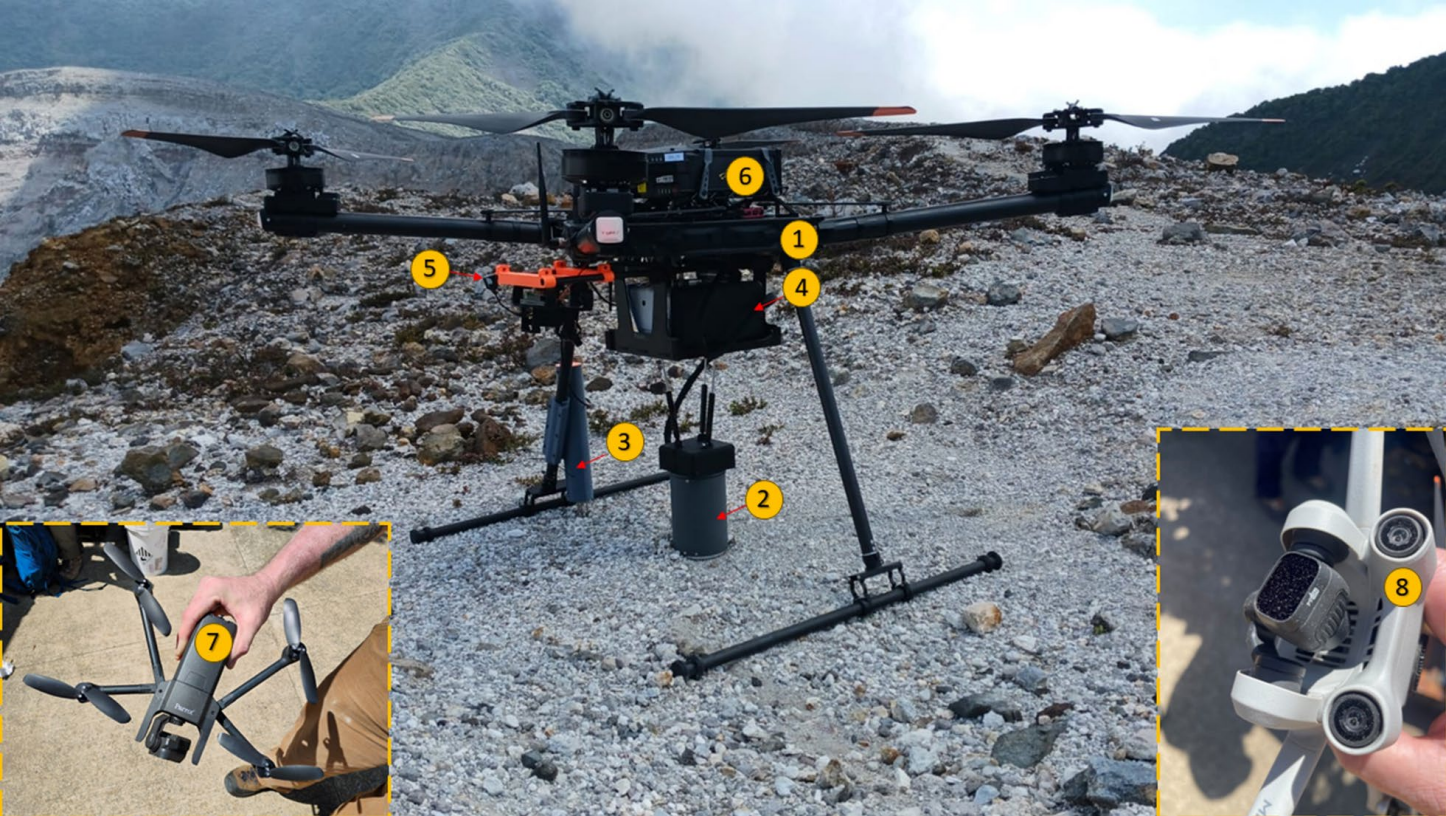
Northern view of the first drone landing site, at the Poás Volcano caldera rim. Labeled equipment includes: (1) Freefly Alta-X Remotely Piloted Aircraft System-RPAS-(“Instrumented RPAS”); (2) SRC-2 Soil Respiration Chamber; (3) Hydra Probe II (Stevens Water Monitoring Systems) for measuring soil moisture and temperature; (4) EGM-5 Portable Gas Analyzer with integrated CO_2_ and H_2_O sensors; (5) 690TVL resolution camera; (6) battery pack; (7) Parrot Anafi drone (“Look-down RPAS”) equipped with a gimballed camera; and (8) DJI Mini 3 Pro drone (“Height RPAS”). The Freefly Alta-X UAV RPAS has a fully deployed rotor-to-rotor diameter of approximately 2.27 m. The deployment of the drone constellation was carried out by a team of three pilots

We developed a customized RPAS for remote soil gas and environmental measurements by integrating multiple sensors onto an unmanned aerial vehicle (Fig. 2). The system included an EGM-5 Portable Gas Analyzer from PP Systems, equipped with an integrated CO_2_ and H_2_O sensor, an SRC-2 Soil Respiration Chamber, and a Hydra Probe II (Stevens Water Monitoring Systems) for measuring soil moisture and temperature. The EGM-5 Portable Gas Analyzer and the SRC-2 Soil Respiration Chamber were mounted beneath a Freefly Alta-X drone. The chamber was securely attached using a system of three nearly vertical gas struts, each exerting a downward gentle force of 22.2 N. This resulted in an equivalent weight of approximately 6.8 kg on the respiration chamber, effectively replicating the conditions during manual soil gas flux measurements. A 690TVL resolution camera connected to a 5.8 GHz analog transmitter was also mounted at the rear of the fuselage of the Freefly Alta-X UAV to provide real-time visual feedback for precise landing and deployment. Additionally, the Hydra Probe II was affixed to one of the drone’s landing legs via a custom-designed spring-loaded attachment. This mechanism ensured that the probe’s four prongs were gently inserted into the soil upon landing, allowing for reliable soil moisture and temperature measurements. The fully equipped Freefly Alta-X, including all attached payloads, had a total mass of approximately 22 kg. All custom attachments for mounting the sensors and instruments were designed and fabricated using 3D printing, allowing for precise integration of the components while maintaining the structural integrity of the RPAS system. This system (“Instrumented RPAS”) was complemented with two additional support RPAS to provide critical visual and positional guidance: one to assist with horizontal positioning and another to provide height references.

The first support RPAS (Parrot Anafi, 0.496 kg), referred to as the “Look-down RPAS”, was equipped with a gimballed camera and stationed directly above the intended landing site. This aircraft hovered at a height of 50 m above the surface, with its camera facing vertically downward. The purpose of this drone was to provide the instrumented RPAS pilot with a clear reference point, ensuring that lateral positioning over the landing site was maintained throughout the approach. The second support RPAS (DJI Mini 3 Pro, 0.290 kg), referred to as the “Height RPAS”, was positioned 3 m above ground level at a horizontal distance of ~15 m from the landing site. Its role was to provide visual cues to help determine the instrumented RPAS’s height during descent. By observing the aircraft from a displaced vantage point, the Height RPAS pilot could provide real-time feedback, guiding the instrumented RPAS safely to the ground.

### Flying protocol to land in a volcano basal area

**Fig. 3:**
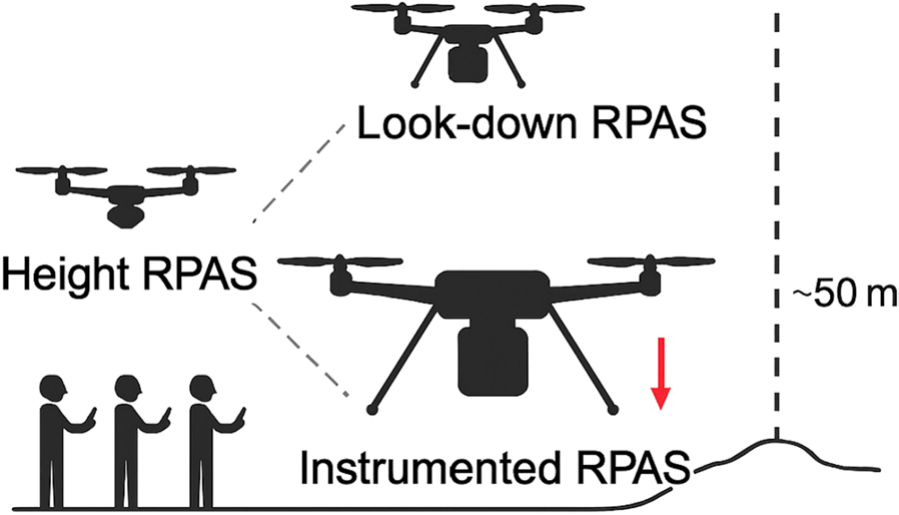
Schematic representation of a coordinated multi-RPAS landing operation conducted at an active volcanic crater. The approach involved three drones: a “Height RPAS” for initial reconnaissance and telemetry acquisition, a “Look-down RPAS” hovering at 50 m to provide visual guidance during landing, and an “Instrumented RPAS” equipped with ground-contact sensors. This panel illustrates the sequential process of landing site verification, aerial positioning, descent coordination, and final touchdown, highlighting the use of real-time visual and telemetry data to ensure a safe and accurate deployment in a complex and hazardous volcanic environment. The deployment of the drone constellation was carried out by a team of three pilots

Landing a Remotely Piloted Aircraft System (RPAS) equipped with sensors that require direct ground contact in a volcano’s basal area presented a series of challenges. These challenges included identifying a suitable landing site, positioning the drone accurately, and ensuring a safe rate of descent and touchdown (Fig. 3). Additionally, the unpredictable nature of the environment, with rapidly changing weather conditions (e.g., shifting winds, fluctuating visibility due to volcanic plumes and cloud cover), further complicated the process.

Before attempting the landing, the “Height RPAS” conducted a reconnaissance of potential landing sites. The objective of this survey was to confirm that the surface was flat and free of significant obstacles that could interfere with a safe touchdown. Additionally, the telemetry data from the “Height RPAS” provided critical information about the horizontal distance and vertical displacement between the takeoff location and the landing site. This data was relayed to the pilots of the other two RPAS, allowing them to adjust their approach accordingly.

Once the site had been verified, the landing sequence began. The “Look-down RPAS” positioned itself directly above the landing site at a height of 50 m with its camera in the nadir position, while the “Instrumented RPAS” initiated its approach. Flying toward the designated area, it descended to 30 m above the surface before continuing forward until it appeared within the “Look-down RPAS” field of view. This visual confirmation allowed the “Instrumented RPAS” pilot to make fine adjustments, ensuring that the aircraft remained precisely aligned with the landing site.

With the aircraft correctly positioned, the descent phase commenced. The “Height RPAS” pilot played a critical role at this stage, providing a countdown as the “Instrumented RPAS” moved from ~30 m down to ~5 m above the surface. At this point, the mission team conducted a final check to ensure that weather conditions and battery levels were suitable for completing the landing.

Following the clearance to proceed, both pilots zoomed in on the descending aircraft to provide higher-resolution visual feedback. Using this enhanced perspective, the “Height RPAS” pilot initiated a final countdown until the “Instrumented RPAS” made contact with the ground. The aircraft remained in place for the designated duration of the data collection process before taking off again and returning to the original takeoff location.

By employing multiple RPAS to provide critical visual and positional references, we were able to successfully execute precise landings in a highly challenging volcanic environment. This approach ensured not only the accuracy of sensor deployment but also the safety of the operation, despite the unpredictable and dynamic conditions of the landing zones.

### Landing targets

**Fig. 4:**
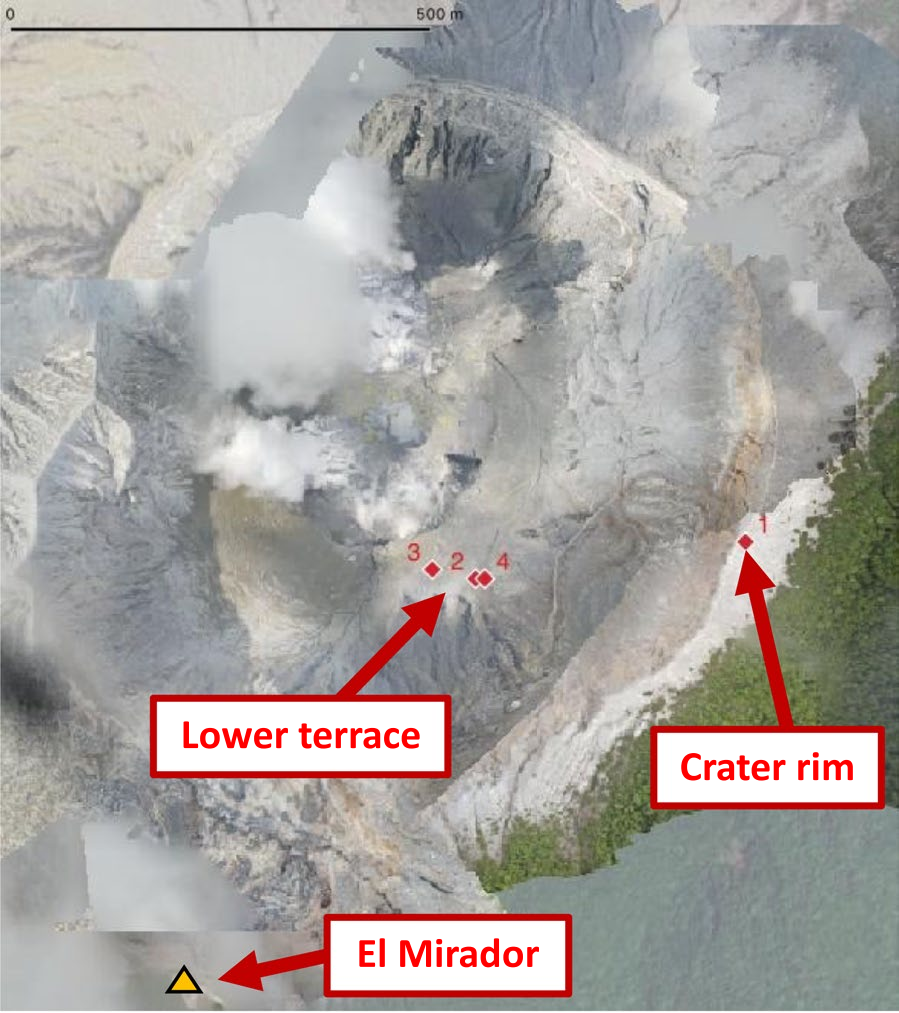
Map showing the four drone landing sites at Poás Volcano. The map was produced using orthomosaics generated from drone imagery, overlaid on satellite imagery obtained via Google Earth Pro. Source: Google Maps; imagery © 2025 Airbus

To ensure optimal locations for soil measurements, landing targets were selected based on a combination of thermal imaging, prior geological knowledge, and RPAS landing feasibility. Thermal imaging was conducted from the “El Mirador” viewpoint using a FLIR T540 camera equipped with a 24° lens to identify anomalously warm areas typically associated with hydrothermal activity and fluid flow (e.g., Neale et al. 2016). To ensure accurate temperature measurements, the FLIR camera was calibrated for ambient temperature (~ 30 °C), relative humidity (~ 50%), and distance to the lower terrace of Poás Crater (~ 700 m). Additionally, local researchers’ expertise and previous reports provided insights into locations with known degassing activity (Epiard et al. 2017; Melián et al., 2019), particularly near outcrops and along caldera walls. Lastly, potential landing sites were evaluated with the “Height RPAS” based on their suitability for safe deployment of the “Instrumented RPAS”. Based on these criteria, we selected four study sites for analysis (Fig. 4):

1. *Crater rim reference site*. One site (Site 1, Fig. 4) was located on the crater rim of Poás Volcano, inside a ballistic impact crater from a previous eruption. This site was easily accessible, allowing a field operator to monitor the experiment in real time. The RPAS-based soil gas measurement was conducted on March 13, 2025. To assess the performance of the RPAS approach, a manual measurement was performed at the same location on the following day using the same instrument. Due to cloud coverage, additional crater rim sites originally planned for manual validation could not be surveyed.
2. *Lower terrace sites*. The remaining three sites (Sites 2–4, Fig. 4) were located on the lower terrace of Poás crater, at around 230 m below the rim observatory (*El Mirador*), an inaccessible and hazardous area due to the volcano’s unrest conditions at the time of the experiment. These sites were selected for their high potential for soil gas emissions and their suitability as landing targets for the Freefly Alta-X UAV. The measurements at these locations aimed to further evaluate the RPAS-based methodology under remote and operationally challenging conditions.

This site selection strategy allowed for both direct comparison with ground-based measurements and an assessment of the RPAS performance in remote, high-risk volcanic environments.

### Measurements

Atmospheric pressure ($P_{{{\text{atm}}}}$) was monitored during the experiment using the PP system’s internal barometer. This parameter was used to identify the RPAS landing and takeoff time, as the pressure time series exhibited a stable plateau during the sampling period in the ground. Additionally, we recorded air temperature ($T_{{{\text{air}}}}$), soil temperature ($T_{{{\text{soil}}}}$), soil moisture ($M_{{{\text{soil}}}}$), carbon dioxide concentration ($C_{{{\text{CO2}}}}$), and water vapor concentration ($C_{{{\text{H2O}}}}$). The latter was derived from absolute vapor pressure, a parameter directly provided by the system. Using these parameters obtained from the sensor measurements collected by the RPAS-mounted instruments, we can determine the ${\text{CO}}_{2}$ and ${\text{H}}_{2}{\text{O}}$ soil fluxes ($\varphi_{{CO_{2} }}$ and $\varphi_{{H_{2} O}}$, respectively) as follows:

1 $$\varphi_{{{\text{CO}}_{{2}} }} = \frac{{{\text{dC}}_{{{\text{CO2}}}} }}{{{\text{dt}}}}\frac{V}{A} \,\,{\text{and}}\,\,\varphi_{{H_{2} O}} = \frac{{{\text{dC}}_{{{\text{H2O}}}} }}{{{\text{dt}}}}\frac{V}{A} ,$$

where $\frac{{{\text{dC}}_{{{\text{CO2}}}} }}{{{\text{dt}}}}$ and $\frac{{{\text{dC}}_{{{\text{H2O}}}} }}{{{\text{dt}}}}$ represent the ${\text{CO}}_{2}$ and ${\text{H}}_{2}{\text{O}}$ assimilation rate, respectively, i.e., the variation of the $CO_{2}$ and ${\text{H}}_{2}{\text{O}}$ concentrations in the accumulation chamber with time $t$; $V$ is the total system volume; and $A$ is the area of soil exposed to the accumulation chamber. For the system used, $V = 0.001171 m^{3}$ and $A = 0.0078 m^{2}$. Over the short measurement period (~10–15 min), we assume the assimilation of ${\text{CO}}_{2}$ and ${\text{H}}_{2}{\text{O}}$ follow a constant flux, resulting in a linear increase in chamber ${\text{CO}}_{2}$ and ${\text{H}}_{2}{\text{O}}$ concentration over time. Ideally, this would produce a constant rate of change in $C_{{{\text{CO2}}}}$ and $C_{{{\text{H2O}}}}$; however, in practice, leakage from the chamber causes a deviation from this ideal behavior, leading to a decrease in the apparent assimilation rate over time. As a result, the expected linear trend may become non-linear, reflecting the influence of leakage on the measurement. To account for these potential leakage effects, we model the ${\text{CO}}_{2}$ and ${\text{H}}_{2}{\text{O}}$ concentrations as linear ($C_{{{\text{CO2}}}} = a_{{{\text{CO2}}}} + b_{{{\text{CO2}}}} t$ and $C_{H2O} = a_{H2O} + b_{H2O} t$) or quadratic ($C_{{{\text{CO2}}}} = a_{{{\text{CO2}}}} + b_{{{\text{CO2}}}} t + c_{{{\text{CO2}}}} t^{2}$ and $C_{H2O} = a_{H2O} + b_{H2O} t + c_{H2O} t^{2}$) functions of time, where $a_{{{\text{CO2}}}}$ and ${\text{H}}_{2}{\text{O}}$ represent initial concentrations in the chamber, $b_{{{\text{CO2}}}}$ and $b_{H2O}$ are the coefficients of the linear term, and $c_{CO2}$ and $c_{H2O}$ capture non-linearities introduced by leakage or other uncontrolled factors. Hence, to estimate the assimilation rate, we define $\frac{{{\text{dC}}_{{{\text{CO2}}}} }}{{{\text{dt}}}} = b_{{{\text{CO2}}}}$ and $\frac{{{\text{dC}}_{H2O} }}{{{\text{dt}}}} = b_{H2O}$ with $b_{{{\text{CO2}}}}$ and $b_{H2O}$ being determined from the linear or quadratic approximation, depending on which approach provides a better fit to the data. This approach allows us to approximate the flux of ${\text{CO}}_{2}$ and ${\text{H}}_{2}{\text{O}}$ into the chamber. After substituting the values of various constants and rearranging factors and units, the ${\text{CO}}_{2}$ flux and ${\text{H}}_{2}{\text{O}}$ flux, expressed in $\left[ {g \cdot m^{ - 2} \cdot d^{ - 1} } \right]$, can be calculated from:

2 $$\varphi_{{{\text{CO}}_{{2}} }} = 0.0687 b_{{{\text{CO2}}}} \frac{{P_{{{\text{atm}}}} }}{{T_{{{\text{air}}}} }},$$

3 $$\varphi_{{H_{2} O}} = 0.0281 b_{{H_{2} O}} \frac{{P_{{{\text{atm}}}} }}{{T_{{{\text{air}}}} }},$$

where $b_{{{\text{CO}}_{{2}} }}$ and $b_{{H_{2} O}}$ are expressed in ${\text{ppm of CO}}_{2} \cdot s^{ - 1}$ and ${\text{ppm of }} H_{2} O \cdot s^{ - 1}$, respectively. All other parameters have been previously defined and must be input in Eqs. (2) and (3) using SI units. Atmospheric pressure ($P_{atm}$) remains approximately constant during the sampling period, fluctuating within the accuracy limits of the pressure sensor. In contrast, air temperature ($T_{air}$) exhibits slight variations, likely due to thermal disequilibrium between the sensors and the surroundings. As a standard criterion, we use the average atmospheric pressure and the average air temperature measured over the last 300 s of the sampling period when applying Eqs. (2) and (3). Moreover, the uncertainty of $\varphi_{{CO_{2} }}$ and $\varphi_{{H_{2} O}}$ at the 95% confidence level $(\sigma_{{\varphi_{CO2} }}$ and $\sigma_{\varphi H2O}$) is estimated using the general rule of error propagation. This calculation considers that the only sources of uncertainty in Eqs. (2) and (3) are $b_{{{\text{CO2}}}}$, $b_{{H_{2} O}}$, $P_{atm}$, and $T_{air}$. Under this assumption, $\sigma_{{\varphi_{{CO_{2} }} }}$ and $\sigma_{H2O}$ can be approximated to:

4 $$\sigma_{{\varphi_{{{\text{CO2}}}} }} = \sqrt {\left( {\frac{{\varphi_{{{\text{CO}}_{{2}} }} }}{{b_{{{\text{CO}}_{{2}} }} }}} \right)^{2} \sigma_{{b_{{{\text{CO}}_{{2}} }} }}^{2} + \left( {\frac{{\varphi_{{{\text{CO}}_{{2}} }} }}{{P_{{{\text{atm}}}} }}} \right)^{2} \sigma_{{P_{{{\text{atm}}}} }}^{2} + \left( {\frac{{\varphi_{{{\text{CO}}_{{2}} }} }}{{T_{{{\text{air}}}}^{2} }}} \right)^{2} \sigma_{{T_{{{\text{air}}}} }}^{2} } ,$$

5 $$\sigma_{\varphi H2O} = \sqrt {\left( {\frac{{\varphi_{{{\text{CO}}_{{2}} }} }}{{b_{{H_{2} O}} }}} \right)^{2} \sigma_{{b_{{H_{2} O}} }}^{2} + \left( {\frac{{\varphi_{{H_{2} O}} }}{{P_{{{\text{atm}}}} }}} \right)^{2} \sigma_{{P_{{{\text{atm}}}} }}^{2} + \left( {\frac{{\varphi_{{H_{2} O}} }}{{T_{{{\text{air}}}}^{2} }}} \right)^{2} \sigma_{{T_{{{\text{air}}}} }}^{2} } ,$$

where $\sigma_{{b_{{{\text{CO}}_{{2}} }} }}$, $\sigma_{{b_{{H_{2} O}} }}$ $\sigma_{{P_{atm} }}$, and $\sigma_{{T_{air} }}$ are the uncertainties of the parameters $b_{{{\text{CO}}_{{2}} }}$, $b_{{H_{2} O}}$, $P_{{{\text{atm}}}}$, and $T_{{{\text{air}}}} ,$ respectively, at 95% confidence level (i.e., twice the standard deviation). $\sigma_{{b_{{CO_{2} }} }}$ and $\sigma_{{b_{{H_{2} O}} }}$ are determined from the best fit to the concentration ($C_{{{\text{CO2}}}}$ and $C_{H2O}$) time series; the uncertainty in atmospheric pressure, $\sigma_{{P_{{{\text{atm}}}} }} ,$ is set to $\pm 0.1 {\text{bar}}$, corresponding to the instrument’s accuracy; and the uncertainty in air temperature, $\sigma_{{T_{air} }}$, is estimated as twice the standard deviation of the air temperatures recorded during the last 300 s of the sampling period or as the thermometer accuracy (± 0.1 °C), whatever is larger. Beyond atmospheric conditions and fluxes, we also retrieved the median soil temperature ($T_{{{\text{soil}}}}$) and the median soil moisture ($M_{{{\text{soil}}}}$) throughout the experiment, with their respective uncertainties defined by the 95% confidence interval.

## Results and discussion

### Site 1: high gas emissions and temperature anomalies

Our flight protocol was successfully executed, allowing us to retrieve measurements from all designated landing sites (Table 1). The drone-based CO₂ flux measurement at Site 1, located at the crater rim, yielded a flux of $\varphi_{{CO_{2} }} = 1.3 \pm 0.6$ $g \cdot m^{ - 2} \cdot d^{ - 1}$. This result is compatible with in situ ground-based measurements taken at the same location ($\varphi_{{CO_{2} }} = 1.41 \pm 0.01$ $g \cdot m^{ - 2} \cdot d^{ - 1}$) on the following day, supporting the reliability of the drone-based methodology. However, a notable difference was observed in the level of uncertainty, with the drone-based measurement exhibiting a significantly higher uncertainty (~ 46%) compared to the ground-based measurement (< 0.01%). We suspect the discrepancy was caused by an incorrect cable connection between the sensors and the EGM-5 Portable Gas Analyzer, as air temperature data were not recorded during the flight, potentially affecting sensor stability.

**Table 1:** Results obtained for the different landing sites

Parameter	Site 1 (UAV)	Site 1 (ground)	Site 2 (UAV)	Site 3 (UAV)	Site 4 (UAV)
Day of the experiment	March 13, 2025	March 14, 2025	March 14, 2025	March 14, 2025	March 14, 2025
Duration of the experiment^*1^ [min]	46.3	10	36.2	29.7	33.6
Analysis time^*2^ [min]	8.8	10	14.5	14.7	14
$P_{{{\text{atm}}}} \left[ {{\text{mbar}}} \right]$	$766.4 \pm 0.1$	$766.5 \pm 0.1$	$776.5 \pm 0.1$	$776.8 \pm 0.1$	$777.0 \pm 0.1$
$T_{air}$[°C]	–	$19.9 \pm 0.1$	$19.2 \pm 1.4$	$21.8 \pm 0.3$	$24.5 \pm 0.3$
$T_{soil}$[°C]	$19.5 \pm 0.4$	$19.2 \pm 0.2$	$17.6 \pm 0.1$	$17.4 \pm 0.1$	$18.9 \pm 0.1$
$M_{soil}$ $\left[ \% \right]$	–	$5.99 \pm 0.07$	–	$1.51 \pm 0.17$	–
$\varphi_{{CO_{2} }}$ $\left[ {g \cdot m^{ - 2} \cdot d^{ - 1} } \right]$	$1.3 \pm 0.6$^*3,4^	$1.41 \pm 0.01$^*4^	0	$0.0210 \pm 0.017$^*5^	–
$\varphi_{{H_{2} O}}$ $\left[ {g \cdot m^{ - 2} \cdot d^{ - 1} } \right]$	$231.8 \pm 4.2$^*3,4^	$51.9 \pm 1.5$^*4^	$4.09 \pm 0.05$^*5^	$1.84 \pm 0.05$^*5^	–

^*1^From the moment the sensor was activated until it was deactivated. ^*2^Analysis time chosen depending on when stability is found**.** ^*3^ $T_{air}$ was not recorded during this experiment due to an electronic misconnection. In this case, $\varphi_{{CO_{2} }}$ and $\varphi_{{H_{2} O}}$ are calculated using $T_{soil}$ as a proxy for ambient temperature. ^*4^ Quadratic fit. ^*5^ Linear fit

**Fig. 5:**
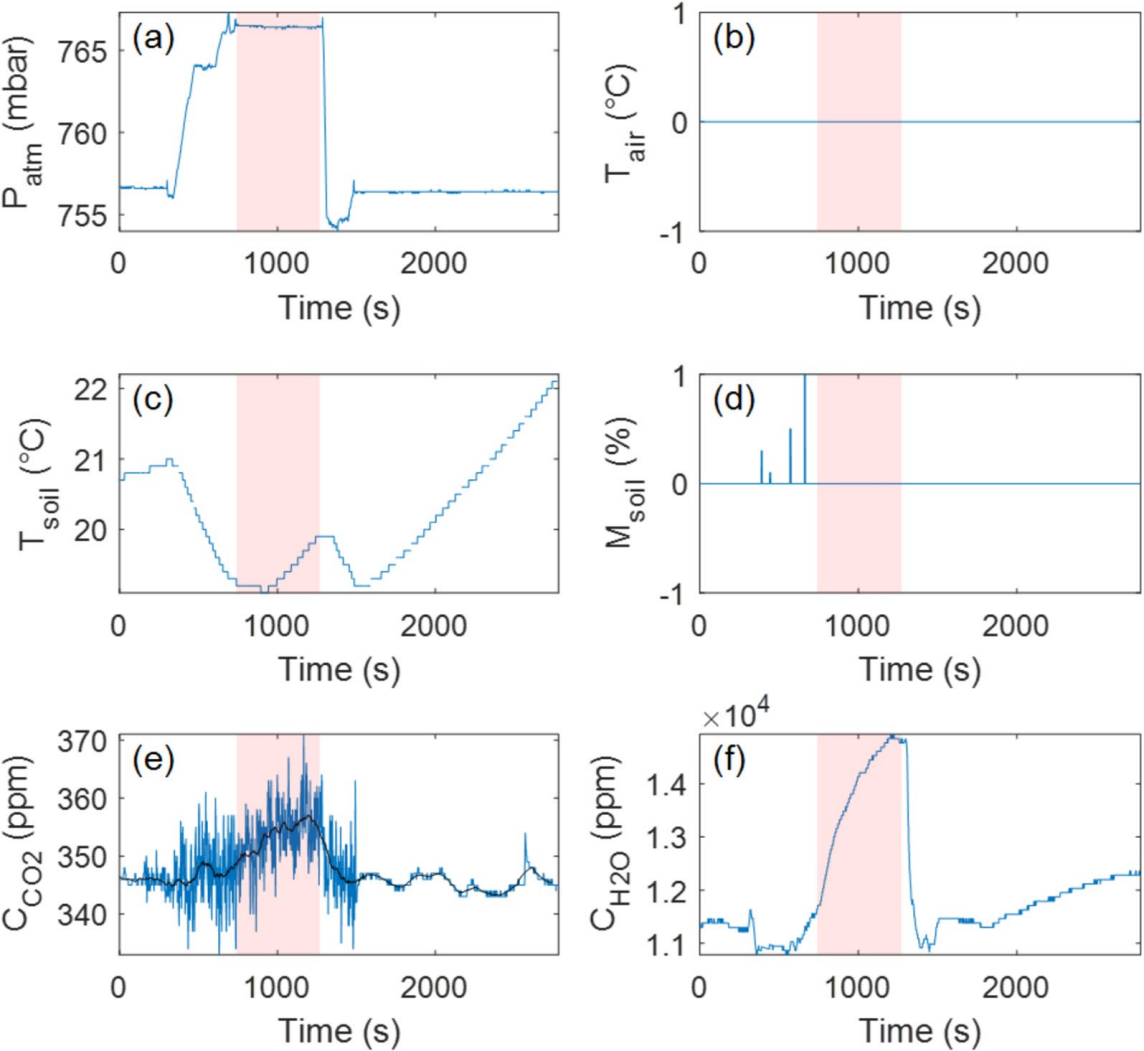
Time series recorded with the Instrumented RPAS at Landing Site 1 (crater rim). **a** Pressure evolution over time, with the flat, high-pressure region corresponding to the period when the Instrumented RPAS remained on the ground for measurements (shown with the pink area). **b** Air temperature inside the accumulation chamber, which in this case was not recorded due to an electronic issue. **c** Soil temperature evolution during the experiment. **d** Soil moisture evolution, though sensor contact with the ground was insufficient for reliable measurements. **e** CO_2_ concentration inside the accumulation chamber over time. The black line is the median filtered signal (100-s window). **f** H_2_O concentration inside the accumulation chamber over time. The rotors in this case stayed active for the first three minutes after landing, yet not change in the trends can be detected

Even with the elevated uncertainty in the drone-based CO_2_ flux ($\varphi_{{CO_{2} }}$), our results consistently indicate that Site 1 (i.e., the crater rim) is the most prominent CO_2_ and H_2_O emitter among all surveyed sites (Fig. 5; Table 1). Interestingly, Site 1 was positioned directly above a known fumarolic zone inside the crater, which might explain the elevated H₂O flux recorded there. Additionally, soil temperature measurements ($T_{{{\text{soil}}}}$) reveal that Site 1 is slightly warmer than Sites 2–4, with a temperature approximately 1.5 °C higher than those recorded at Sites 2 and 3. Differences in soil temperature may be related to the timing of the measurements; however, it is worth noting that subtle thermal anomalies were also recorded along the crater rim during a concurrent, ground-based soil degassing campaign (García-Martínez et al., *manuscript submitted*).

**Fig. 6:**
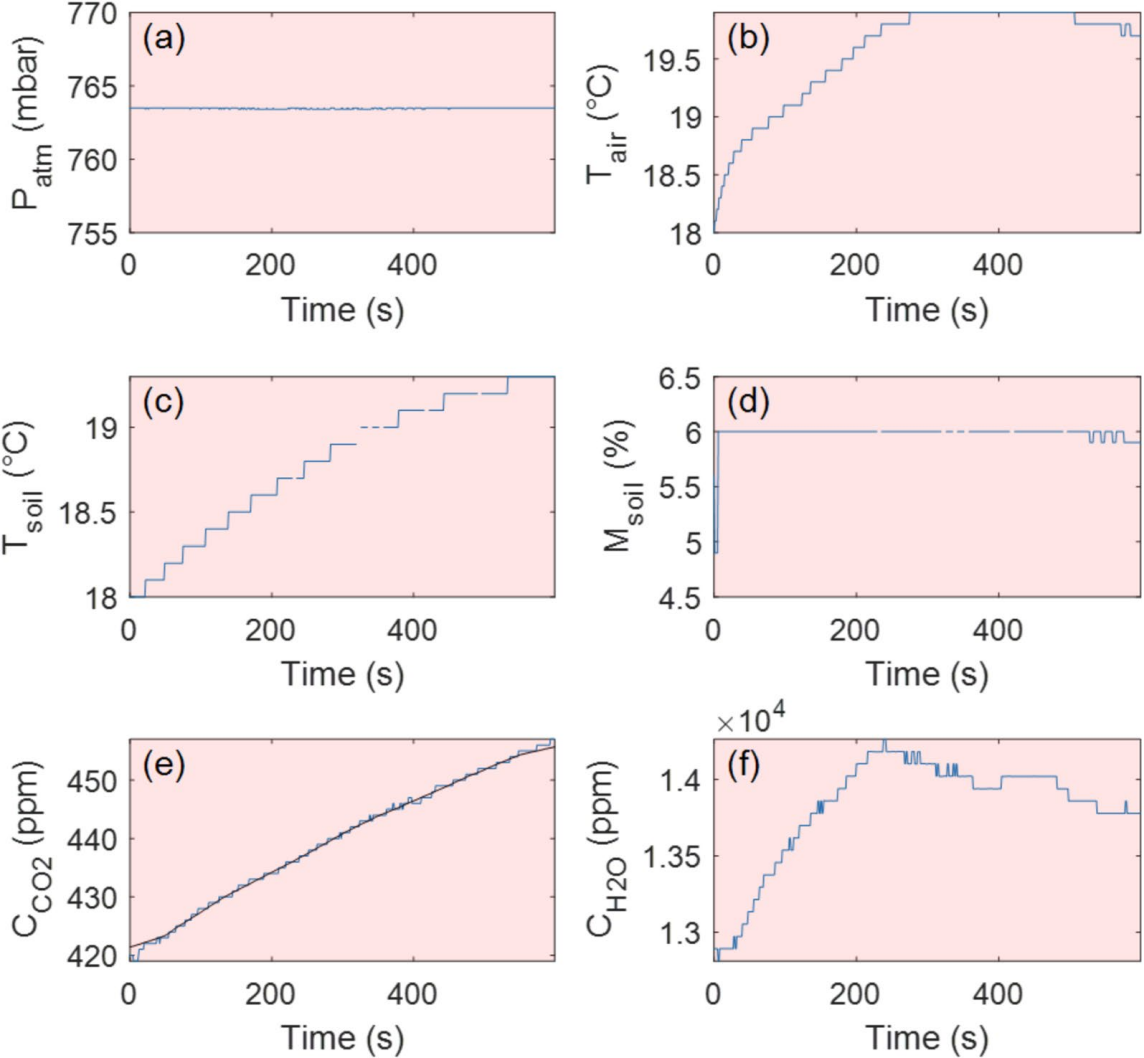
Time series recorded manually (for reference) at Landing Site 1 (crater rim). **a** Pressure evolution over time. **b** Air temperature inside the accumulation chamber. **c** Soil temperature evolution during the experiment. **d** Soil moisture evolution. **e** CO_2_ concentration inside the accumulation chamber over time. The black line is the median filtered signal (100-s window). **f** H_2_O concentration inside the accumulation chamber over time. This experiment was performed with no detectable trend change despite RPAS rotors remaining active for the first three minutes before being turned off

Interestingly, a strong discrepancy was observed in H_2_O flux ($\varphi_{{H_{2} O}}$) measurements between the drone-based and ground-based datasets (Figs. 5 and 6; Table 1). The underlying cause of this inconsistency remains unclear but may be attributed to natural variations in evapotranspiration due to shifting environmental and/or volcanic conditions. Notably, the drone-based measurement at Site 1 was conducted one day earlier than the ground-based measurement, suggesting that daily variations in moisture fluxes could contribute to the observed differences. The electronic noise that likely affected the CO_2_ measurements at this site may have also influenced the H_2_O sensor readings, potentially complicating direct comparisons. However, the uncertainty levels for $\varphi_{{H_{2} O}}$ remained consistent between drone-based and ground-based measurements (~ 2–3%), suggesting that the H_2_O data were not significantly impacted by any electronic issues. Hence, we conclude that the measurable H_2_O flux likely implies the presence of active near-surface moisture transport. This could reflect shallow hydrological circulation, maybe influenced by the subsurface condensation of magmatic volatiles and percolation through fractured substrate (e.g., Girona et al. 2021; Zhan et al. 2022); however, H_2_O fluxes should be interpreted with caution, as their dependence on atmospheric conditions remains uncertain.

### Site 2: weak H_2_O flux and no CO_2_ emissions

**Fig. 7:**
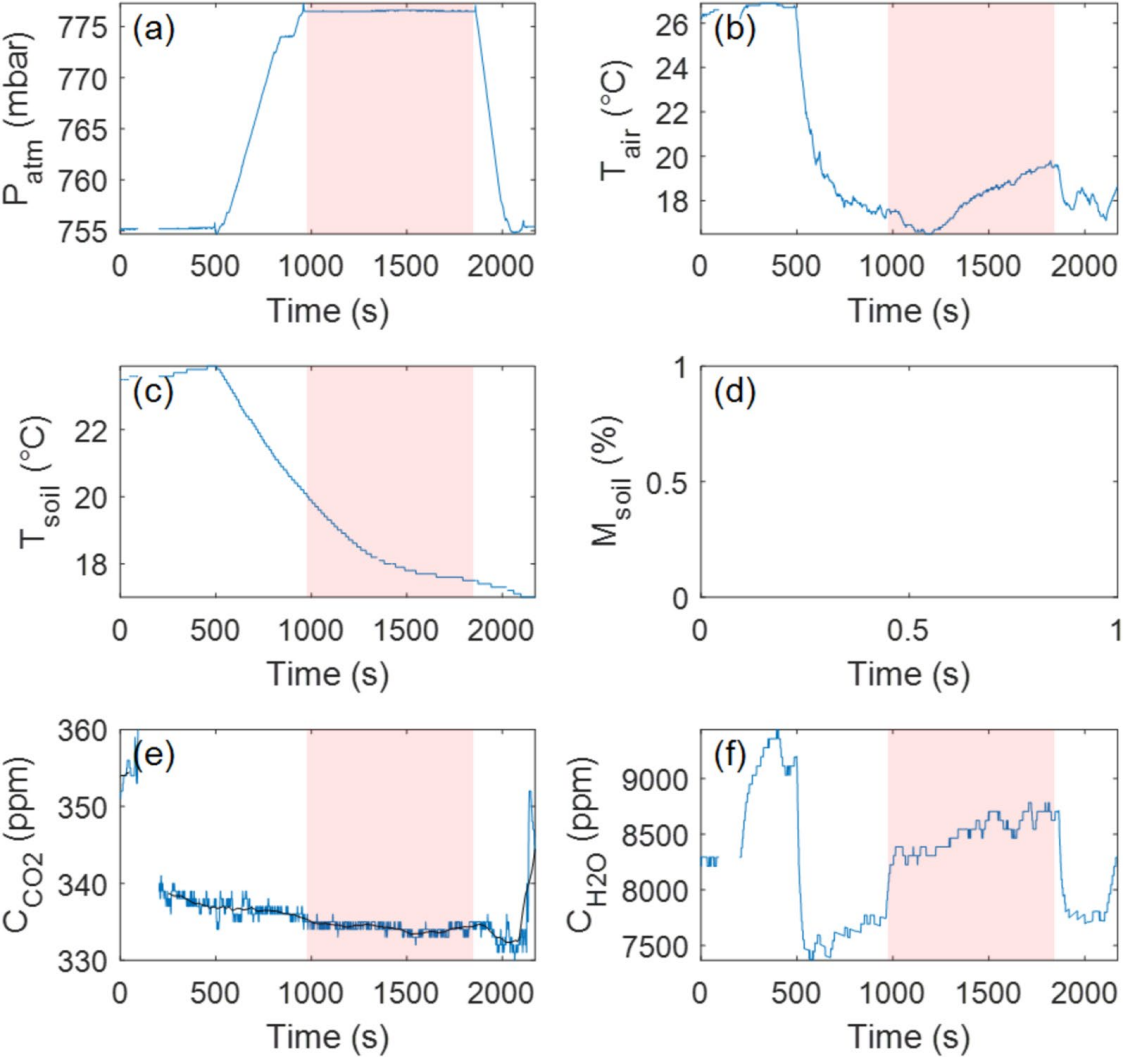
Time series recorded with the Instrumented RPAS at Landing Site 2 (lower terrace). **a** Pressure evolution over time, with the flat, high-pressure region corresponding to the period when the Instrumented RPAS remained on the ground for measurements (shown with the pink area). **b** Gas temperature inside the accumulation chamber. **c** Soil temperature evolution during the experiment. **d** Soil moisture evolution, though sensor contact with the ground was insufficient for reliable measurements. **e** CO_2_ concentration inside the accumulation chamber over time. The black line is the median filtered signal (100-s window). **f** H_2_O concentration inside the accumulation chamber over time

At Site 2, our measurements revealed significantly lower H_2_O fluxes than those observed at the crater rim, yet higher fluxes ($\varphi_{{H_{2} O}} = 4.09 \pm 0.05$ $g \cdot m^{ - 2} \cdot d^{ - 1}$) compared to other locations on the lower terrace (Fig. 7; Table 1). Moreover, no detectable CO_2_ emissions were observed at this site, which may be attributed to a lack of CO_2_ supply from depth, the absence of open permeable pathways, or interaction with shallow subsurface water. In the latter case, significant H_2_O fluxes may enhance CO_2_ scrubbing, preventing it from reaching the surface (unless the shallow water in the terrace is acidic, which we consider unlikely). Finally, soil moisture data were not obtained at this site, likely due to insufficient penetration of the Hydra Probe II sensor prongs, which resulted in inadequate contact with the soil.

### Site 3: most successful drone-based experiment

**Fig. 8:**
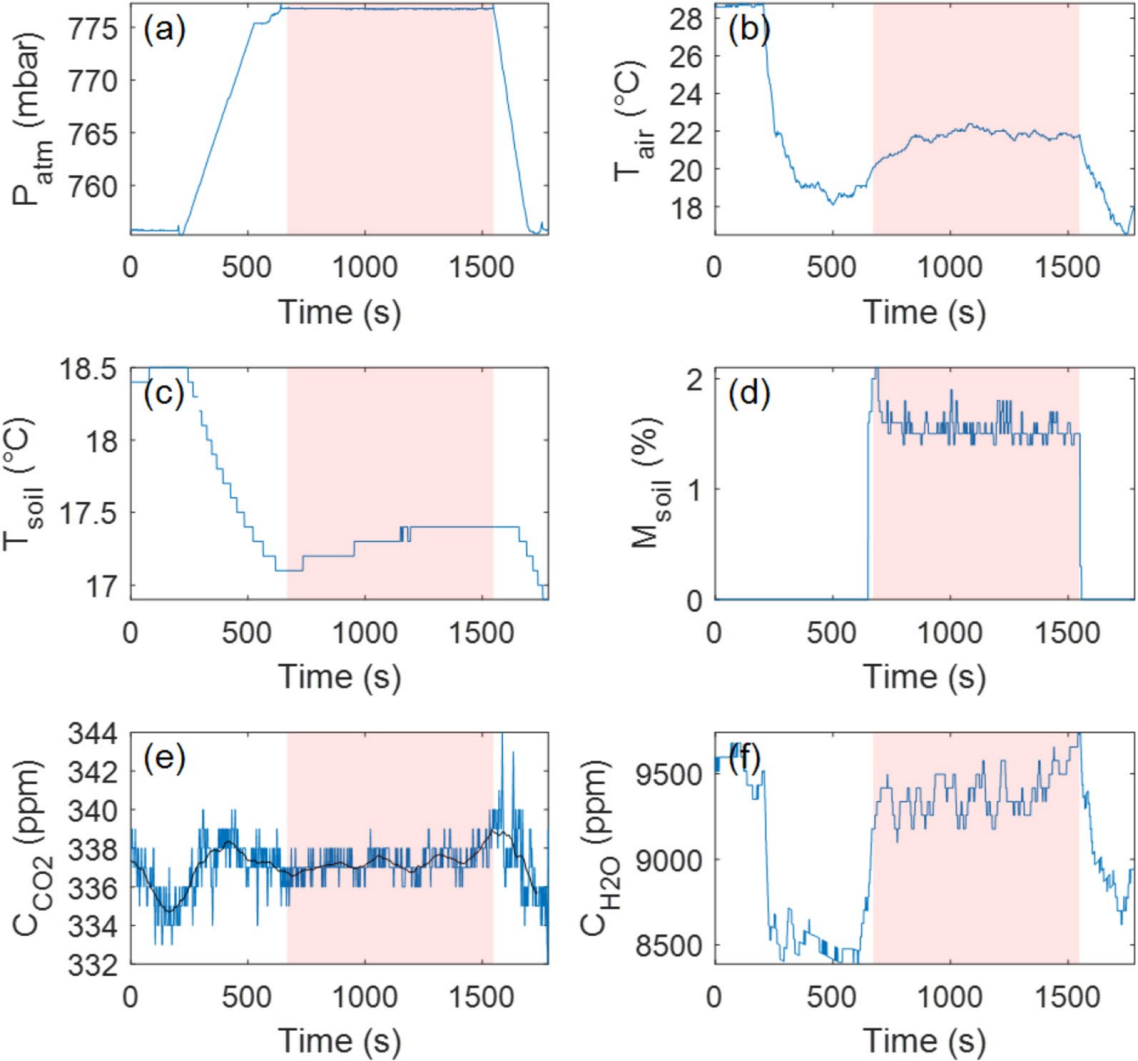
Time series recorded with the Instrumented RPAS at Landing Site 3 (lower terrace). **a** Pressure evolution over time, with the flat, high-pressure region corresponding to the period when the Instrumented RPAS remained on the ground for measurements (shown with the pink area). **b** Air temperature inside the accumulation chamber. **c** Soil temperature evolution during the experiment. **d** Soil moisture evolution, though sensor contact with the ground was insufficient for reliable measurements. **e** CO_2_ concentration inside the accumulation chamber over time. The black line is the median filtered signal (100-s window). **F** H_2_O concentration inside the accumulation chamber over time

Site 3 was the only location where the drone-based measurement was fully operational and yielded a complete dataset without technical issues (Fig. 8; Table 1). The experiment lasted approximately 30 min, with an active analysis period of 14.7 min. Soil temperature at this site reached 17.4 ± 0.1 °C, and soil moisture was recorded at 1.51 ± 0.17%. Both CO_2_ and H_2_O fluxes were weak yet statistically significant at the 95% confidence level, with values of $\varphi_{{{\text{CO}}_{{2}} }}$ = 0.0210 ± 0.017 ${\text{g }} \cdot {\text{m}}^{ - 2} \cdot {\text{ d}}^{ - 1}$ and $\varphi_{{H_{2} O}} =$ 1.84 ± 0.05 ${\text{g }} \cdot {\text{m}}^{ - 2} \cdot {\text{ d}}^{ - 1}$. The low CO_2_ and H_2_O fluxes suggest limited magmatic, biogenic, or hydrothermal degassing activity in this area, possibly indicating that this sector of the lower terrace is not currently acting as a preferential pathway for deep gas escape.

### Site 4: evidence of a sealing issue and volcanic activity influence

**Fig. 9:**
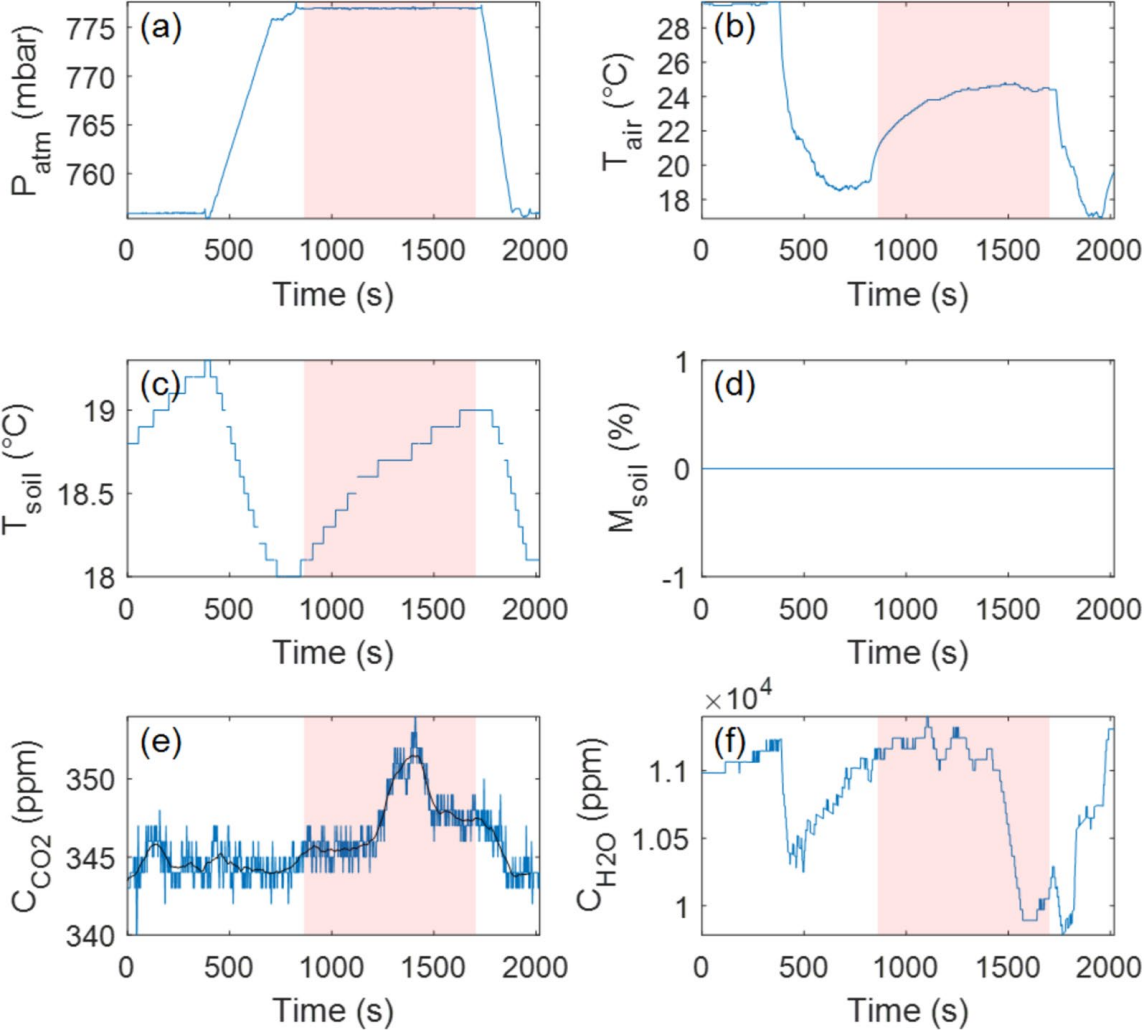
Time series recorded with the Instrumented RPAS at Landing Site 4 (lower terrace). **a** Pressure evolution over time, with the flat, high-pressure region corresponding to the period when the Instrumented RPAS remained on the ground for measurements (shown with the pink area). **b** Air temperature inside the accumulation chamber. **c** Soil temperature evolution during the experiment. **d** Soil moisture evolution, though sensor contact with the ground was insufficient for reliable measurements. **e** CO_2_ concentration inside the accumulation chamber over time. The black line is the median filtered signal (100-s window). **f** H_2_O concentration inside the accumulation chamber over time

At Site 4, our measurements suggest a probable sealing issue with the accumulation chamber, as suggested by the observed pattern of CO_2_ concentration changes (Fig. 9; Table 1). Specifically, a sudden increase (of up to ~7 ppm) in CO_2_ concentration inside the chamber was followed by a rapid decrease (of up to ~5 ppm), which is inconsistent with the expected gas accumulation behavior. One possible explanation for this pattern is the passage of a volcanic plume through the site, as the volcano was highly active on the day of the experiment (March 14, 2025). The simultaneous decrease (over 100 ppm) in H_2_O concentration further supports this hypothesis, suggesting that external atmospheric disturbances may have affected gas exchange dynamics within the chamber. These observations highlight the importance of ensuring proper chamber sealing and accounting for external environmental influences when interpreting drone-based gas flux data.

### Comparison with ground-based data from other studies

During our field experiment, Poás Volcano exhibited low-level eruptive activity, including minor phreatic explosions. Persistent gas-and-steam emissions were observed, with volcanic plumes containing elevated concentrations of sulfur dioxide (SO₂), hydrogen sulfide (H₂S), and tephra (Global Volcanism Program 2025). These emissions contributed to the dynamic and hazardous conditions at the summit area.

Soil gas and temperature measurements were performed at two key locations: the crater rim and the lower terrace. At the crater rim, both soil temperature and CO_2_ fluxes were low, consistent with values previously reported during periods of volcanic unrest (2000–2003; Melián et al., 2019) and quiescence (2012; Epiard et al. 2017). These low values thus appear to be characteristic of the area, where weak endogenous degassing is often overlapped with biological CO_2_ production in the soil. In contrast, previous studies have shown that the lower terrace is generally associated with higher soil temperatures and CO_2_ fluxes (Epiard et al. 2017; Melián et al., 2019), with spatial variability linked to proximity to active fumaroles. However, measurements from our study indicate that the sites instrumented in this campaign were located outside or at the margins of the most active degassing zones. This spatial setting explains the moderate-to-low CO_2_ fluxes recorded in the lower terrace, despite its historically higher degassing potential. In any case, although our dataset is not sufficient to estimate the total diffuse CO_2_ output from Poás in 2025, the observed low fluxes align with the most recent soil degassing survey by Epiard et al. (2017), which reported a total emission of 0.9 ± 0.5 t·d^-1^. In addition to site selection, other factors may have contributed to the low CO₂ fluxes observed, including near-surface sealing of permeable pathways caused by the deposition of fine hydrothermal ash. Furthermore, RPAS access limitations prevented landings in steep, rocky terrains known to host more vigorous diffuse and fumarolic emissions.

Unlike CO_2_, soil flux of H_2_O and soil moisture are seldom measured during routine volcanic field campaigns, and to our knowledge, this study represents the first report of H_2_O flux at a volcano. As a result, opportunities for direct comparison with previous investigations remain limited. Despite this, our study supports the value of incorporating H_2_O flux and soil moisture measurements alongside CO_2_ in soil degassing surveys. The joint analysis of both gases may enhance our ability to detect and characterize variations in shallow fluid migration processes, which are often associated with episodes of volcanic unrest (e.g., Jasim et al. 2019). Additionally, H_2_O flux and soil moisture are expected to reflect shallow H_2_O circulation, influenced by both crustal processes (e.g., vapor condensation, subsurface flow) and surface environmental factors such as rainfall, evapotranspiration, and soil type. In hydrologically active systems like Poás, H_2_O flux and soil moisture may provide useful context for interpreting degassing patterns. For example, high soil moisture may indicate active subsurface H_2_O fluxes that enhance CO_2_ scrubbing, potentially suppressing surface emissions, while low soil moisture could suggest drier, more permeable conditions that favor gas escape. Given the likely non-linear and site-specific nature of these relationships, further investigation is needed to assess the diagnostic power of soil H_2_O flux and soil moisture in volcanic monitoring, particularly in wet environments such as Poás.

### Challenges and potential improvements

Despite some technical challenges, our experiments demonstrate the viability of using drone-based platforms for soil gas flux measurements in volcanic environments. However, several key areas require improvement to enhance the accuracy, reliability, and operational efficiency of this technique.

One major challenge was identifying suitable landing spots and ensuring proper ground preparation for flux measurements. Uneven terrain, loose pebbles, ash, and volcanic debris can compromise the chamber’s sealing against the ground, leading to measurement errors. A more advanced instrumented RPAS equipped with a surface preparation mechanism (such as a small air blower or mechanical brush) could help clear debris and ensure a proper seal between the chamber and the soil. Additionally, incorporating a high-precision height detection system would facilitate more controlled and stable landings, reducing disturbances to the measurement process.

Chamber sealing remains a crucial aspect of ensuring accurate flux measurements. Potential leaks or external gas interferences (such as transient volcanic plumes) may have influenced recorded concentrations (above all in Site 4), introducing variability in the results. A more robust chamber design with adaptive sealing mechanisms, such as flexible skirts or an inflatable tire, could significantly reduce leaks and improve the consistency of measurements.

An additional challenge was the altered flight dynamics of the drones in the high-altitude volcanic environment. The most noticeable effect was the ‘mushy’ handling of the aircrafts, characterized by reduced maneuverability. The drones exhibited a greater tendency to be affected by wind gusts and atmospheric turbulence compared to operations at lower altitudes. This instability is due to the lower air density, which reduces the efficiency of the propellers and diminishes overall aerodynamic control. Additionally, sudden wind shifts near the crater and along steep terrain further exacerbated these handling issues, making precise landings and steady hovering more difficult. Future deployments could benefit from adjustments to flight control algorithms, increased thrust capabilities, or the use of advanced stabilization systems to improve the Instrumented RPAS performance in such challenging conditions.

By addressing these key technical challenges, including the improvement of landing stability, refinement of ground preparation techniques, optimization of chamber sealing, and enhancement of maneuverability at high altitudes, drone-based soil degassing measurements may evolve into a robust and reliable approach for volcanic gas monitoring. Although not intended to replace established ground-based surveys, drone-based surveys may provide valuable complementary information by enabling data acquisition in areas that are otherwise inaccessible due to safety constraints. In this way, the RPAS approach contributes to bridging the gap between traditional field-based methods and emerging remote sensing technologies, ultimately enhancing our capacity to monitor and understand volcanic activity from a safer distance and a broader observational perspective.

## Conclusions

This study provides a proof of concept, demonstrating the feasibility and potential of employing Remotely Piloted Aircraft Systems (RPAS) to quantify local soil CO_2_ and H_2_O emissions, and to characterize other ground properties within active volcanic environments. By deploying a drone-based accumulation chamber system, we obtained measurements at four locations of varying accessibility and degassing activity around the Poás Volcano crater. Among these, Site 1, located at the crater rim, exhibited the highest CO_2_ and H_2_O emissions, confirming it as the primary degassing zone within the study area. Site 2, situated on a terrace inside the crater, showed the highest H_2_O flux in that area, despite an absence of detectable CO_2_, suggesting localized hydrological contributions without significant magmatic gas release. Site 3 yielded the most complete and technically successful dataset, allowing for confident interpretation of both fluxes under stable operational conditions. In contrast, Site 4 presented evidence of potential interference from volcanic activity, underscoring the challenges of data acquisition and interpretation in highly dynamic environments.

Although technical limitations were encountered, particularly related to ground contact and electronic noise, these did not preclude the extraction of meaningful data trends. Our experiments highlight the importance of improving field protocols, such as automated ground preparation and chamber sealing, to enhance measurement reliability. More broadly, our study reveals the spatial heterogeneity of soil degassing across small-scale topographic and structural variations in the active crater. By enabling access to hazardous or previously inaccessible zones, drone-based methods offer a powerful tool to detect subtle soil degassing patterns and refine our understanding of gas and fluid migration in active volcanic systems. Future work will benefit from further integrating RPAS platforms with multi-sensor packages and real-time telemetry, enhancing the technique’s capabilities for soil monitoring and hazard assessment in volcanic regions.

## Supplementary Information

Additional file 1.

Additional file 2.

Additional file 3.

Additional file 4.

Additional file 5.

## Data Availability

The data supporting the findings of this study are available with this manuscript as supporting files.
